# Immunotherapy with check-point inhibitors (CPI) in adult malignancies: a protocol for the systematic review of the quality of economic analyses

**DOI:** 10.1186/s13643-019-1047-z

**Published:** 2019-06-11

**Authors:** Ying Wang, Pierre Camateros, Denise Smith, David Dawe, Peter Ellis

**Affiliations:** 1 BC Cancer Vancouver, 600 West 10th Ave, Vancouver, British Columbia V5Z 4E6 Canada; 2 Department of Medicine, Division of Community Internal Medicine, University of British Columbia, 2775 Laurel Street, 10th Floor, Vancouver, British Columbia V5Z 1M9 Canada; 3 Department of Medical Oncology, CancerCare Manitoba, 675 McDermot Ave, Winnipeg, Manitoba R3E 0V9 Canada; 4 Faculty of Health Sciences, Health Science Library, McMaster University, 1280 Main St W, Hamilton, Ontario L8S 4L8 Canada; 5 Department of Oncology, McMaster University, Juravinski Cancer Centre, 3rd floor, 699 Concession Street, Hamilton, Ontario L8V 5C2 Canada

**Keywords:** Immuno-therapy, Immune-oncology, Check-point inhibitor, Economic analysis, Cost-effectiveness, Cancer, Neoplasm, Quality, Reporting quality, Protocol, CHEERS criteria

## Abstract

**Background:**

Immuno-oncology, and in particular, check-point inhibitors (CPIs), have led to a paradigm shift in the field of cancer care. The cost of new drug development is high, and many novel agents in oncology are significantly more expensive than older agents. Therefore, healthcare funders have factored measures of cost-effectiveness into decisions concerning drug reimbursement and incorporation of new agents into treatment algorithms. The methodology of cost-effectiveness evaluations, however, is less rigorously applied than those evaluating clinical efficacy and safety data. Thus, in spite of many regulatory bodies having approved CPIs based on existing economic analyses, to date, there has not been a systematic evaluation of the quality of health economic studies conducted on this new class of agents.

Therefore, we propose to systematically review the methodologic and reporting quality of cost-effectiveness and cost-utility studies assessing CPIs to alternate established therapies, other immuno-oncology regimens, or placebo, in adults with malignancies.

**Methods/design:**

The systematic review will include all published economic evaluations of CPIs compared with at least one other treatment in adult patients with solid or hematologic malignancies. A search will be performed to identify relevant studies in Ovid MEDLINE, EMBASE, Cost-effectiveness Analysis Registry, Evidence-Based Medicine Reviews, and the NIHR-HTA database. The titles and abstracts of all identified studies will be independently reviewed by two reviewers, who will then assess the full text of all articles deemed to meet eligibility criteria. Assessed articles will be screened for compliance with the Consolidated Health Economic Evaluation Reporting Standards (CHEERS) criteria. The association, with CHEERS criteria, of the journal impact factor, publication year, funding source, tumor site, trial or model-based study, and CPIs studied, will then be assessed.

**Discussion:**

The systematic review will aim to provide an overview of the quality of economic analyses evaluating CPIs for the treatment of malignancies in adult patients. Any systemic or recurrent deficiencies in methodological or reporting quality will be described and used to inform recommendations for improved reporting of economic analyses.

**Systematic review registration:**

This review will not be registered with PROSPERO, it does not meet the eligibility criterion of addressing an outcome of the direct patient or clinical relevance.

**Electronic supplementary material:**

The online version of this article (10.1186/s13643-019-1047-z) contains supplementary material, which is available to authorized users.

## Background

### Rationale

Immuno-oncology has led to a paradigm shift in the field of cancer care. Since its inception, this new class of systemic cancer therapy has received at least 26 Food and Drug Administration (FDA) approvals across 17 different cancer types, with 940 more agents being studied in clinical development as of September 2017 [1].

Contrary to traditional cytotoxic chemotherapy, CPIs enhance the body’s immune system to induce antitumor activity [2]. This allows for comparatively more tolerable adverse events and increased efficacy in certain tumor types. CPIs have been integrated within the treatment pathways of many solid and hematologic malignancies, such as lung and head and neck cancer, urothelial and renal cell carcinoma, and lymphoma. Furthermore, CPIs are under development for use in many more tumor types.

Since healthcare in countries such as Canada and the UK is administered under a publicly funded system, governments have insisted that, prior to approval for funding, new therapies must not only demonstrate clinical effectiveness, but also cost-effectiveness as well. Countries without single-payer systems are also facing the need to account for the rising costs of care. The American Society of Clinical Oncology (ASCO) has recently focused attention on the importance of considering financial impacts in addition to other criteria when assessing new therapies [3]. Thus, along with clinical effectiveness, evaluations of the economic effectiveness of immunotherapies, and in particular check-point inhibitors (CPIs), is of importance.

Unlike the validated approaches for the assessment of clinical effectiveness, such as randomized controlled trials, the assessment of cost-effectiveness is currently not as rigorously scrutinized. Some analyses combine real-world patient-level data with quality of life data collected from randomized controlled trials in single disease sites [4]. Other studies combine multiple disease sites across different countries with inherently different perspectives and healthcare payment systems [5, 6]. Decision-makers often use incremental cost-effectiveness ratios (ICERs) to compare new immunotherapy regimens with existing therapies; however, this method is nuanced and plagued by inaccuracies, such that experts in the field are calling into question the true validity of such analyses [7]. Others have proposed the addition of a standardized scale for grading financial toxicity, parallel to the Common Terminology Criteria for Adverse Events, a system commonly used to evaluate new agents in clinical trials [8].

In an attempt to standardize health economic studies, the International Society of Pharmacoeconomics and Outcomes Research (ISPOR) has developed an aggregate guideline for authors and reviewers to take into consideration. The Consolidated Health Economic Evaluation Reporting Standards (CHEERS) Statement was developed by international experts, and jointly endorsed by 10 journals including the British Medical Journal in 2013 [9, 10].

Previous studies have adopted the CHEERS checklist as their reference for evaluating the quality of published health economic studies in multiple settings [11–13]. In particular, the quality of economic analyses of oral cancer drugs [14], multiple myeloma therapies [15], and adjuvant breast cancer radiotherapy [16], have been assessed in this way. These studies have revealed that adherence to the CHEERS standards was quite variable ranging from 50% to nearly complete compliance.

Although many regulatory bodies have approved immunotherapies based on existing economic analyses, there has not been a review of the quality and reporting of health economic studies conducted on this new class of agents.

### Objectives

Thus, we propose to systematically review the methodologic and reporting quality of cost-effectiveness and cost-utility studies comparing new CPIs used in immune-oncology regimens, including anti-PD1 agents (nivolumab and pembrolizumab), anti-PDL1 agents (atezolizumab, avelumab, and durvalumab), and the anti-CTLA4 agents (ipilimumab and tremelimumab), to established therapies in adults with solid and hematologic malignancies.

## Methods

### Protocol and registration

This review will not be registered with the international prospective register of systematic reviews (PROSPERO) as it does not meet the eligibility criterion of addressing an outcome of direct patient or clinical relevance.

This protocol was developed based on the Preferred Reporting Items for Systematic review and Meta-Analysis Protocols (PRISMA-P) 2015 checklist (Additional file 1: Figure S1) [17, 18], and any protocol amendments will be described, dated, and a rationale provided.

### Inclusion and exclusion criteria

Only studies meeting the following criteria will be included:

- Articles reporting on the results of economic analyses with cost-effectiveness, cost-utility, or cost-benefit analyses. This includes pre-planned analyses within randomized control trials, meta-analyses, or any study design using appropriate economic modeling. Articles focusing on the methodological aspects of economic analyses or reporting only on cost-of-illness or budget impacts will be excluded.
- Articles comparing at least two treatment arms, one of which could be placebo, in adults (at least 18 years old or older, or if children were included, where the data for treatment in adults is separately reported and analyzed) with hematologic and solid malignancies, where at least one treatment arm used one of the following immunotherapeutic agents, which were approved at the time of study design:
  - ○Anti-PD1 agents (nivolumab and pembrolizumab)
  - ○Anti-PDL1 agents (atezolizumab, avelumab, and durvalumab)
  - ○Anti-CTLA4 agents (ipilimumab and tremelimumab)
- Articles published in English between 1 January 2007 and 31 December 2018, inclusively. This period was chosen as it captures all reports on the above-mentioned therapeutic agents in human trials.
- Full text and original articles. Abstracts, conference proceedings, editorials, letters, reviews, and systematic reviews will be excluded. In the event of multiple publications reporting an analysis of the same underlying data, all articles meeting the inclusion criteria will be included in the analysis given the focus on methodological and reporting quality, rather than the conclusion reached by the underlying studies.

### Information sources, data management, and study selection

A systematic search will be performed in Ovid MEDLINE® (1946 to present), OVID Embase (1974 to present), Cost-effectiveness Analysis Registry (1926 to 2015), Evidence-Based Medicine Reviews, and NIHR-HTA (1989 to present) with the search strategy detailed in Table 1. Literature search results will be uploaded to EPPI-Reviewer 4 [19], in which two reviewers will independently assess the titles and abstracts of all articles retrieved from the database search. The full-text of articles potentially meeting the inclusion criteria will then be assessed for inclusion by the same two independent reviewers. Neither author will be blinded to the authors, institution, or journal of publication. Any disagreements will be resolved through discussion and consensus between the two reviewers, and a third reviewer will be asked to adjudicate on any cases where consensus cannot be reached. Data will then be extracted from all full-text articles selected for analysis according to the data extraction form detailed in Table 2.

**Table 1:** Search strategy

Search term	
1. Immunotherapy/	
2. immunotherap*.ti,ab.	
3. immuno therap*.ti,ab.	
4. immuno-oncology.ti,ab.	
5. immunooncology.ti,ab.	
6. immuno oncology.ti,ab.	
7. immuno-therap*.ti,ab.	
8. chemoimmunotherap*.ti,ab.	
9. chemo-immunotherap*.ti,ab	
10. checkpoint inhibitor*.ti,ab.	
11. check-point inhibitor*.ti,ab.	
12. check point inhibitor*.ti,ab.	
13. check-point block*.ti,ab.	
14. checkpoint block*.ti,ab.	
15. check point block*.ti,ab.	
16. check-point therap*.ti,ab.	
17. checkpoint therap*.ti,ab.	
18. check point therap*.ti,ab.	
19. pd-l1.ti,ab.	
20. pd-1.ti,ab.	
21. ctla-4.ti,ab.	
22. exp CTLA-4 Antigen/	
23. Cytotoxic T-Lymphocyte Associated Antigen 4.ti,ab.	
24. Cytotoxic T-Lymphocyte Antigen 4.ti,ab.	
25. exp Programmed cell death 1 receptor/	
26. Programmed cell death 1 receptor.ti,ab.	
27. Programmed cell death 1 protein.ti,ab.	
28. Pd 1 protein.ti,ab.	
29. PDCD1 protein.ti,ab	
30. CD279 antigen.ti,ab.	
31. nivolumab.ti,ab.	
32. Opdivo.ti,ab.	
33. pembrolizumab.ti,ab.	
34. Keytruda.ti,ab.	
35. durvalumab.ti,ab.	
36. Imfinzi.ti,ab.	
37. tremelimumab.ti,ab.	
38. Exp ipilimumab/	
39. Yervoy.ti,ab.	
40. atezolizumab.ti,ab.	
41. Tecentriq.ti,ab.	
42. avelumab.ti,ab.	
43. Bavencio.ti,ab.	
44. Economics/	
45. exp Costs and Cost Analysis/	
46. Economics, Medical/	
47. Economics, Nursing/	
48. Economics, Pharmaceutical/	
49. exp Economics, Hospital/	
50. exp “Fees and Charges”/	
51. exp Budgets/	
52. economic*.ti,ab.	
53. cost*.ti,ab.	
54. pric*.ti,ab.	
55. fees.ti,ab.	
56. pharmacoeconomic.ti,ab.	
57. pharmaco-economic.ti,ab.	
58. expendit*.ti,ab.	
59. (value adj2 mone*).ti,ab.	
60. exp models, economic/	
61. exp Neoplasms/	
62. cancer*.ti,ab.	
63. neoplas*.ti,ab.	
64. tumor.ti,ab.	
65. tumor.ti,ab.	
66. malignan*.ti,ab.	
67. or/1–43	
68. or/44–60	
69. or/61–66	
70. 67 and 68 and 69

**Table 2:** Data extraction form

Variable name	Allowable values	Explanation
Baseline trial characteristics		
ID	Valid PubMed ID number	Pubmed ID
Journal	Name of journal	Journal of publication
Year	2007–2018	Year of publication
Tumor site	Non-small cell lung cancer Melanoma Renal cell carcinoma Squamous cell carcinoma of the head and neck Urothelial carcinoma Classical Hodgkin lymphoma Other	The tumor type for which treatment with a check-point inhibitor was analyzed.
Check_point_1	Nivolumab	Name of the first check-point inhibitor being compared.
	Pembrolizumab	
	Atezolizumab	
	Avelumab	
	Durvalumab	
	Ipilimumab	
	Tremelimumab	
Check_point_2	Nivolumab	Name of 2nd check-point inhibitor being compared, if applicable.
	Pembrolizumab	
	Atezolizumab	
	Avelumab	
	Durvalumab	
	Ipilimumab	
	Tremelimumab	
Comparator_1	Placebo	Name of the 1st non-check-point-inhibitor agent or chemotherapeutic regimen being compared, if applicable.
	Name of other agent or regimen	
Comparator_2	Placebo	Name of the 2nd non-check-point-inhibitor agent or chemotherapeutic regimen being compared, if applicable.
	Name of other agent or regimen	
Funding	Industry/government	Funding source declared by article
Country	List of country names	List of all the countries in which the economic analysis applied as reported by the article.
Multiple_countries	Y/N	Did the economic analysis apply to multiple countries?
CHEERS criteria		
Title	C/I	As detailed in CHEERS recommendations [9]
Abstract	C/PC/I	As detailed in CHEERS recommendations [9]
Background & Objectives	C/PC/I	As detailed in CHEERS recommendations [9]
Target population	C/PC/I	As detailed in CHEERS recommendations [9]
Setting_Location	C/PC/I	As detailed in CHEERS recommendations [9]
Perspective	C/PC/I	As detailed in CHEERS recommendations [9]
Comparators	C/PC/I	As detailed in CHEERS recommendations [9]
Time horizon	C/PC/I	As detailed in CHEERS recommendations [9]
Discount rate	C/PC/I	As detailed in CHEERS recommendations [9]
Health outcomes	C/PC/I	As detailed in CHEERS recommendations [9]
Effectiveness	C/PC/I	As detailed in CHEERS recommendations [9]
Preference for outcomes	C/PC/I/NA	As detailed in CHEERS recommendations [9]
Resources and cost	C/PC/I	As detailed in CHEERS recommendations [9]
Resource quantities	C/PC/I	As detailed in CHEERS recommendations [9]
Choice of model	C/PC/I	As detailed in CHEERS recommendations [9]
Assumptions	C/PC/I	As detailed in CHEERS recommendations [9]
Analytic methods	C/PC/I	As detailed in CHEERS recommendations [9]
Study parameters	C/PC/I	As detailed in CHEERS recommendations [9]
ICERs	C/PC/I	As detailed in CHEERS recommendations [9]
Uncertainty	C/PC/I	As detailed in CHEERS recommendations [9]
Heterogeneity	C/PC/I/NA	As detailed in CHEERS recommendations [9]
Limitations	C/PC/I	As detailed in CHEERS recommendations [9]
Funding source	C/I	As detailed in CHEERS recommendations [9]
Conflict of Interest	C/I	As detailed in CHEERS recommendations [9]

*C* complete; *PC* partially complete; *I* incomplete; *NA* not applicable

### Synthesis of results

A qualitative description of the quality of included articles will be performed and data extracted (Table 2) will be provided in text and table form. All extracted data will be presented by article in table form. A random effects model will be used to assess the association of journal impact factor, year of publication, funding source, tumor site, trial or model-based study, and check-point-inhibitor studied on compliance with CHEERS criteria. The significance, magnitude, and confidence interval of associations will be presented in table form. Significance will be set at a two-sided *p* value of ≤ 0.05. All data will be analyzed using STATA 14 (StataCorp. 2015. Stata Statistical Software: Release 14. College Station, TX: StataCorp LP). As the current study focuses on the quality and reporting of economic analyses of CPIs, rather than the reported results, and the high heterogeneity expected of the included studies, no attempts will be made to quantitatively synthesize or analyze the results of the underlying studies, nor the risk of bias of the results, of the included articles. Wherever possible, all results will be presented in accordance with the PRISMA guidelines [20, 21].

## Discussion

The described systematic review will aim to provide an overview of the quality of economic analyses on the use of novel check-point inhibitor therapies for the treatment of malignancies in adult patients. Any systemic or recurrent deficiencies in methodological or reporting quality will be described, and barriers to improvement explored, in an attempt to better characterize the quality of the evidence base on which financial toxicity assessments and funding policies are determined.

## Additional file

Additional file 1: PRISMA-P 2015 checklist. (DOCX 33 kb)

## Data Availability

The datasets generated and/or analyzed during the current study are available from the corresponding author upon reasonable request.

## Abbreviations

ASCO: American Society of Clinical Oncology
CHEERS: Consolidated Health Economic Evaluation Reporting Standards
CPIs: Check-point inhibitors
CTLA4: Cytotoxic T-lymphocyte-associated protein 4
FDA: Food and Drug Administration
ICER: Incremental cost-effectiveness ratio
ISPOR: International Society for Pharmacoeconomic and Outcomes Research
NIHR-HTA: National Institute for Health Research–Health Technology Assessment
PD1: Programmed death-1
PDL1: Programmed cell death ligand
PRISMA: Preferred Reporting Items for Systematic review and Meta-Analysis
PRISMA-P: Preferred Reporting Items for Systematic review and Meta-Analysis Protocols
PROSPERO: International prospective register of systematic reviews
